# Traditional Chinese Medicine in Treating Children With Coronavirus Disease 2019: A Scoping Review

**DOI:** 10.3389/fped.2022.935551

**Published:** 2022-07-19

**Authors:** Naifan Duan, Bin Liu, Xiaona Li, Yibai Xiong, Li Li, Yan Ma, Cheng Lu

**Affiliations:** Institute of Basic Research in Clinical Medicine, China Academy of Chinese Medical Science, Beijing, China

**Keywords:** COVID-19, children, traditional Chinese medicine, syndrome differentiation, efficacy and safety

## Abstract

Coronavirus disease 2019 (COVID-19) is currently widely spread across the world. Traditional Chinese Medicine (TCM) plays an important role in the overall treatment process. As a special group of population, the treatment outcome of children with COVID-19 has attracted much attention. Our study summarizes the current situation of TCM treatment of children with COVID-19. The results showed that TCM displayed a positive role in the treatment process, and that no significant adverse reactions were found. Our findings provide analytical evidence for the efficacy and safety of TCM participation in the treatment of COVID-19 in children.

## Introduction

In the past 2 years, coronavirus disease 2019 (COVID-19) has repeatedly emerged as an acute respiratory infectious disease. Children have their own unique physiological and pathological characteristics, and they respond differently to the virus compared with adults. Studies have shown that patients with mild COVID-19 may be less likely to be seroconverted in children than in adults at the same viral load (1). It has also been shown that long-term humoral immune responses to severe acute respiratory syndrome coronavirus 2 (SARS-CoV-2) infection in children last longer even after asymptomatic infection than in adults (2). At present, the emergence of SARS-CoV-2 variants has caused the increasing prevalence in children around the world (3–6). More research is urgently needed on the long-term relationship between COVID-19 and children (7). Clinicians have tried a variety of therapeutic regimens and constantly summarized the experience during the process. Corresponding treatment guidelines have been formulated around the world. Among the regimens, Traditional Chinese Medicine (TCM) is mentioned in diagnosis and treatment plans in China (8–11), and it is widely used and shows unique advantages. In March 2022, the World Health Organization (WHO) Expert Meeting on Evaluation of TCM in the Treatment of COVID-19 was held, and it was noted that participation of TCM could reduce the aggravation rate of mild and moderate patients and shorten the duration of viral shedding and hospital stay. The safety of TCM treatment is similar to that of conventional treatment (12). Currently, most studies have focused on describing the TCM efficacy evidence of COVID-19 in adults, and there is less evidence about children.

Studies have shown that children with COVID-19 have a variety of initial symptoms, some have fever and respiratory symptoms (13, 14), some have digestive tract symptoms (15, 16), and some patients have no obvious clinical symptoms but only have fatigue (17). In the course of disease development, there are more changes in symptoms. TCM can flexibly respond to changes in symptoms according to syndrome differentiation. Children act as a special group of people, and a comprehensive and systematic evaluation of the efficacy of TCM is an urgent problem to be solved at present. The results can provide a reference for doctors to guide drug use and for the formulation of a TCM prevention and treatment of COVID-19 policy for children. Our study summarizes the current situation of TCM treatment of COVID-19 in children.

## Methods

### Search Strategy

The following databases were searched from establishment to 2 December 2022: CNKI, Wanfang, SinoMed, PubMed, Cochrane Library, and Embase. The MeSH terms include COVID-19, children, and TCM. We also manually searched for studies that met our inclusion criteria from other sources that were not included in the aforementioned databases. Two researchers (Duan N. F. and Liu B.) independently selected eligible studies. Studies in any language were retrieved.

### Inclusion and Exclusion Criteria

The inclusion criteria were as follows: (1) meeting the diagnostic criteria of COVID-19 diagnosis and treatment protocol: version 4–8 (18–22), (2) randomized controlled trials (RCTs), retrospective cohort studies, and retrospective clinical observational studies, (3) patient age < 18 years old, (4) the intervention measures are integrated Traditional Chinese and Western medicine or TCM alone, and (5) the primary outcome is clinical outcome, and the secondary outcomes can include with or without hospitalization, time to viral shedding, adverse reactions, and time to symptom resolution. The exclusion criteria were as follows: (1) guidelines, reviews, network pharmacology, and basic experimental research, (2) suspected case research, (3) no treatment regimens were described, and (4) missing primary outcome data.

### Data Extraction and Risk of Bias Assessment

According to standard information extraction tables, two researchers (Duan N. F. and Liu B.) independently extracted the data. Throughout the process, disagreements were resolved by discussion or by involving another researcher (Lu C.). The basic information extracted from the articles included authors’ names, publication year, published region, type of study design, date of illness onset, virus detection results, number of cases, sex, age, medical history, epidemiological history, type of clinical classification, syndrome differentiation, symptoms, tongue image, treatment regimens, course of treatment, outcome indicators, and adverse reactions.

We conducted a risk of bias assessment of the included RCT studies. Two reviewers (Duan N. F. and Li X. N.) independently assessed the risk of bias in each study using the criteria outlined in the Cochrane Handbook (2019). Any disagreements were resolved by discussion or by involving another author (Lu C.). The risk of bias was assessed according to the following domains: (1) random sequence generation, (2) attrition bias, (3) allocation concealments, (4) blinding of participants and personnel, (5) blinding of outcome assessment, (6) incomplete outcome data, (7) selective outcome reporting, and (8) other biases. Each potential source of bias was graded as high, low, or unclear, providing a quote from the study report and a justification of our judgment in the “risk of bias” table. In the table, red represents high risk, yellow represents unclear risk, and green represents low risk. We also added notes in the table when information on the risk of bias was related to unpublished data or correspondence with a trial author. When evaluating treatment effects, we considered the risk of bias in studies that contributed to the outcome.

### Data Synthesis and Analysis

The Review Manager 5.2 software was used to produce the risk of bias summary figure. Categorical variables were expressed as counts and percentages. Continuous variables were described using median with interquartile range. All the statistical analyses were performed using SAS version 9.4 (SAS Institute Inc.).

## Results

### Search Results and Study Characteristics

**FIGURE 1:**
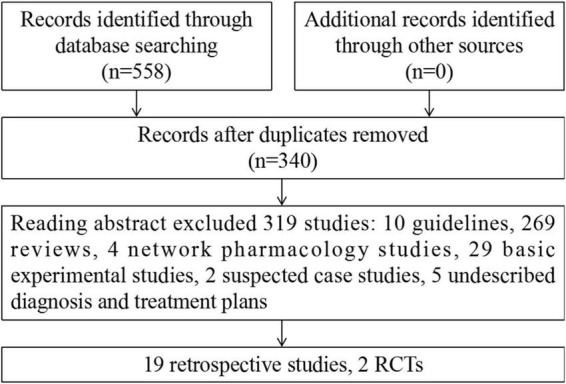
Flowchart of including and excluding studies.

A total of 558 studies were identified using the search strategy and included 72 from CNKI, 121 from Wanfang, 91 from SinoMed, 134 from PubMed, 0 from the Cochrane Library, and 140 from Embase. Of these, 218 duplicate studies were excluded, and 319 studies were excluded after abstract review. Ultimately, 21 studies involving 406 cases were included. Among these, there were 19 retrospective studies (23–41) and 2 RCTs (42, 43). The flowchart of the screening process is presented in Figure 1.

### Retrospective Clinical Study

Twelve studies were published in 2020 and 7 in 2021. The distribution area covers 13 provinces in China. The age range is mainly 7–18 years old. There were 147 men and 99 women. Clinical classification was 13 asymptomatic, 133 mild, and 81 moderate types. The main symptoms were fever and cough, and some cases were accompanied by nausea and vomiting, diarrhea, constipation, and other gastrointestinal symptoms. Another part of the cases did not show clinical symptoms during the medical process. A very small number of children had a previous medical history. Detailed characteristics of the studies are presented in Table 1.

**TABLE 1:** Characteristics of 19 retrospective studies on children with COVID-19.

Study	Sex		Age (Y)	Medical history	Type			TCM syndrome	Symptoms	TCM			Western medicine			Outcomes			Dis charged
	M	F			Asymptomatic	Mild	Moderate			Decoctions	CPMs	Others	Antiviral drugs	Antibiotics	Others	Period (D)	Fever resolution (D)	Viral shedding (D)	
Ge et al. (23); Hubei	0	1	12.5	−	0	0	1	D-H syndrome, L-S Qi-Yin deficiency syndrome	Fever, cough, headache, chest tightness, heavy body, poor appetite, loose stool	Sanren Decoction, Shenling Baizhu Powder, Maxing Shigan Decoction, Yupingfeng Powder	−	−	IFN, Ribavirin	Ceftezole	Defervescence	15	−	−	Yes
Yuan et al. (24); Fujian	50	37	8.4 (3–13)	G6PD deficiency1, Surgery of ASD repair 1, Hyperthyroidism 1, Chronic tonsillitis 1,Allergic rhinitis 1	0	49	38	D-H syndrome	No symptoms 24, fever 59, chill 3, sweating 3, nasal congestion 14, runny nose 9, headache 2, cough 22, sore throat 2, throat itching 1, dry mouth 5, hyposphraesia 1, hypogeusia 2, poor sleep 3, constipation 12 and diarrhea 1	Shangjiao Xuanbi Decoction, Shengjiang Powder, Maxing Shigan Decoction, Shashen Maidong Decoction, Shengmai Powder	−	Herbal plaster andgargle	−	−	Symptomatic support treatment	15.9	1.76	−	Yes
Hu et al. (25); Jilin	11	10	10 (1.6–17)	−	2	13	6	D-H syndrome, C-D syndrome	No symptoms 11, cough 6, fever7, diarrhea 2, bitter mouth 1, pharyngeal discomfort 1, and nasal congestion and runny nose 1	Hanshiyi Decoction, Hanshi Zufei Decoction, QFPD Decoction, Xuanfei Baidu Powder, Wenfei Huashi Decoction, Jieji Qingre Decoction, Pingwei Powder, Wenbu Pishen Decoction	−	Xi yanping, Xuebijing Injection	Arbidol, Ribavirin	Ceftriaxone sodium	Anticoagulation, Oxygen inhalation	−	−	16 ± 7.19	Yes
Liu et al. (26); Shanxi	0	2	8 (7–9)	−	1	1	0	C-D syndrome	Cough phlegm	QFPD Decoction	−	−	IFN, LPV/r	−	−	9.5	−	−	Yes
Luo et al. (27); Henan	0	3	6 (0.5–8)	−	3			−	−	TCM Decoction	−	−	IFN, LPV/r	−	−	−	−	−	Yes
Shang et al. (28); Shanghai	1	0	7	−	0	0	1	D-H syndrome, L-S Qi-Yin deficiency syndrome	Fever, chills, light cough, nausea	Huangqin Qingre Lishi Mixture, Jinbai Mixture	Xiaoer Chaigui Tuire Granule, Pudilan Oral liquid, Xingpi Yanger Granule, Huaiqihuang Granule, Huangqi Granule	−	IFN	−	Triviable bifidobacteria	21	−	−	Yes
Chen et al. (29); Hubei	3	4	8 (2.6–14)	2 cases	2	0	5	−	Fever 4, cough and sputum 5	TCM Decoction	−	−	Arbidol	−	−	−	−	−	Yes
Chen et al. (30); Hubei	25	7	6.9 (0.2–15.1)	Surgery of ASD repair 1, Ophthalmic strabismus surgery 1, ALL 1	0	30	2	Yidu Yufei Syndrome	Fever, chill, cough, muscle soreness, headache, chest tightness, sore throat, vomiting, abdominal pain, poor appetite, diarrhea, convulsions	TCM Decoction	-	−	IFN, Oseltamivir	Azithromycin	Gamma globulin	−	−	−	Yes
Si et al. (31); Guizhou	1	0	13	−	0	1	0	C-D syndrome	−	Mahuang Jiazhu Decoction, Huoxiang Zhengqi Powder, Shengjiang Powder	−	−	−	−	Symptomatic support treatment	13	−	−	Yes
Yang et al. (32); Yunnan	4	1	7.5 ± 5.2	−	2	2	1	−	Cough 1, pharyngeal discomfort 1, myalgia 1	QFPD Granule, Qingyun Jiere Decoction, Xuanfei Baidu Decoction	−	−	−	−	Symptomatic support treatment	15	−	15.2 ± 4.4	Yes Yes
	7	4	9.8 ± 5.2	Hepatitis B 1	1	7	3	−	Fever 5, cough 3, fatigue 1, hyposphraesia or hypogeusia 1, nasal congestion 1, runny nose 2, pharyngeal discomfort 6		−	−	−	−		32	−	32.6 ± 7.0	
Ji et al. (33); Hubei	3	1	0.9 (0.76–10)	−	0	1	3	−	Fever 2, cough 1, nasal congestion 1, short of breath 1	Maxing Shigan Decoction, Shegan Mahuang Decoction, Xiaochaihu Decoction, Wuling Powder	−	−	IFN, Arbidol, Oseltamivir	Azithromycin	Human immune globulin, Splenamino peptide, Budesonide, Magnesium isogglycyrrhiza, Prototype glutathione, High-dose vitamin C	15	−	−	Yes
Zhan and Bai (34); Hubei	3	3	8.5 (0.5–11)	−	1	4	1	−	Fever 4, cough 3, sore throat 3, sneezing 1	TCM Decoction	BairuiGranule, LHQW Capsule, Fufang Yuxingcao syrup	Tanreqing Injection	IFN	Potassium amoxicillin-clavulanate, Piperacillin sodium tazobactam, Cephalosporins, Norvancomycin, Azithromycin	Sodium fructose diphosphate, Gammaglobulin, Ibuprofen, Oxygen inhalation, Atomization, Adjustment of intestinal flora	−	−	1–6	Yes
Tian et al. (35); Tianjin	2	0	9.5 (9–10)	−	0	0	2	Shiwen Syndrome 2, Shixie Kunbiao Syndrome 1, Fengwen Xibiao Syndrome 1	Fever 2, cough 2, fatigue 1, epistaxis 1, dry stool 1	Huoxiang Zhengqi Powder, Yinqiao Powder, Maxing Shigan Decoction, Sanren Decoction	−	−	IFN	−	−	−	−	12.5	Yes
Hu et al. (36); Hunan	7	2	3.9 ± 3.7	−	0	9		−	Fever, cough, headache, dizziness, poor tolerance	COVID-19 Prevention 2 Decoction	LHQW Capsule	−	IFN, Paramivir, Albidol, Oseltamivir, LPV/r	Ceftazidime	Methyl prednisolone, Human immunoglobulin	10.8 ± 6.5	−	−	Yes
Chen et al. (37); Guangzhou	1	0	7	−	1	0	0	−	Asymptomatic	Zhengyang Decoction, QFPD Decoction, Toujie Quwen Decoction	−	−	IFN, LPV/r, Ribavirin	Cefixime, Azithromycin	−	−	−	23	Yes
He et al. (38); Shanghai	6	5	5.5 (1.8–10)	Hepatic insufficiency after BA 1	0	6	5	Yidu Fanwei Syndrome, D-H syndrome, L-S Qi-Yin deficiency syndrome	Early stage: fever 7, cough 9, nasal congestion and runny nose 4; middle stage: cough 5, poor appetite 1, loose stool 1	Huangqin Qingre Lishi Mixture, Shegan Mixture, Jinbai Mixture	Pudilan oral liquid	−	IFN, Ribavirin	Azithromycin	Ibuprofen, Ambroterol, Bifidobacteria	10–29 (19.2 ± 7.2)	−	7–27(14.7 ± 7.2) (nasopharynx); 8–26(15.5 ± 7) (Feces)	Yes
Zhang et al. (39); Yunnan	2	2	6.42 (3–10)	−	1	2	1	Xiefan Shaoyang Syndrome	Fever 3, cough 3, sore throat 1, fatigue 2	Xiaochaihu Decoction	−	−	IFN, LPV/r, Ribavirin	Antibiotics	−	−	−	4–10 (case 1, 2, 3); 21 (case4)	Yes
Cao et al. (40); Shandong	20	17	0–18	−	2	8	11	−	Fever 13, cough 7, diarrhea 1, chills 1, headache 1, nasal congestion 2, runny nose 2, sore throat 1, vomiting 2, abdominal pain 1, constipation 1	TCM Decoction	LHQW Capsule	Tanreqing Injection	IFN, Oseltamivir, Ribavirin, LPV/r	−	Propoglobulin plus coenzyme Q10 was combined with vitamin C	−	−	7–23	Yes
Liu et al. (41); Hubei	1	0	1.92	−	0	0	1	−	Diarrhea	TCM Decoction	−	−	-	−	Symptomatic support treatment	21	−	19	Yes

*“M,” male; “F,” female; “Y,” year; “D,”, day; “TCM,” Traditional Chinese Medicine; “CPMs,” Chinese patent medicines; “G6PD deficiency,” glucose-6-phosphate dehydrogenase deficiency; “Surgery of ASD repair,” surgery of atrial septal defect repair; “ALL,” acute lymphoblastic leukemia; “Hepatic insufficiency after BA,” hepatic insufficiency after biliary atresia; “D-H syndrome,” damp-heat syndrome; “L-S Qi-Yin deficiency syndrome,” Lung/spleen Qi-Yin deficiency syndrome; “C-D syndrome,” cold-damp syndrome; “IFN,” interferon; “LPV/r,” Lopinavir/Ritonavir; “LHQW Capsule,” Lianhua Qingwen capsule; “QFPD Decoction,” Qingfei Paidu decoction; “−,” not mentioned in the original study.*

#### Treatment Regimens Analysis

Fifteen studies used integrated TCM and Western medicine treatment regimens, and 4 studies used TCM treatment regimens alone. TCM treatment is based on syndrome differentiation according to different case characteristics. The cases of 6 studies had damp-heat syndrome, 3 showed cold-damp syndrome, and 3 had lung/spleen Qi-Yin deficiency syndrome in the later stage of the disease. The remaining studies did not clearly specify the type of syndrome differentiation. The main symptoms of damp-heat syndrome include fever, cough, headache, chest tightness, heavy body, poor appetite, loose stool, reddened tongue, and yellow greasy tongue coating. The main symptoms of cold-damp syndrome include fever, cough, nasal congestion, runny nose, pale tongue, and white greasy tongue coating. The main symptoms of lung/spleen Qi-Yin deficiency syndrome include fever, cough, fatigue, pharyngoxerosis, reddened tongue, and thin tongue coating.

The most commonly used type of drug is TCM decoction. Cold-damp syndrome uses *Qingfei Paidu Decoction* more often. If the damp-heat syndrome is more damp than heat, *Huoxiang Zhengqi Powder*, *Sanren Decoction*, *Shenling Baishu Powder* are used, if the heat is more than damp, *Maxing Shigan Decoction* will be added. For the Lung-spleen Qi-Yin deficiency syndrome, the most commonly used medicine is *Yupingfeng Powder*, *Shashen Maidong Decoction*, and *Shengmai Powder*. The commonly used Chinese patent medicines include *Lianhua Qingwen capsule*, *Xiaoer Chaigui Tuire granules*, *Pudilan Xiaoyan oral liquid*, etc. A part of the cases used TCM injection for adjuvant treatment.

Western medicine treatment regimens are mainly antiviral drugs combined with antibiotics and symptomatic supportive treatment according to disease situation. Among the 19 studies included, the number of antiviral drug use times is: 13 studies using interferon, 6 studies using Ribavirin, 4 studies using Arbidol, 6 studies using Lopinavir/Ritonavir, and 4 studies using Oseltamivir. The number of antibiotic use times is: 5 studies using azithromycin, 3 studies using cephalosporin antibiotics, 2 studies using penicillinase antibiotics, and 1 study using vancomycin. The number of immunotherapy use times is: 3 studies using gamma globulin and 1 study using human immune globulin. The symptomatic support treatment programs include hormonotherapy, regulating intestinal flora, defervescence, anticoagulation, oxygen inhalation, etc. The details are presented in Table 1.

#### Treatment Outcomes

The clinical outcomes of all the cases included in the 19 studies were cured and discharged. Ten studies counted the length of hospital stay, which ranged from 9.5 to 32 days. Among them, there were 6 studies using integrated traditional Chinese and Western medicine regimens, and the length of hospital stay was 15, 9.5, 21, 15, 10.8, and 19.2, respectively. There were 4 studies using TCM regimens alone, and the length of hospital stay was 15.9, 13, 15-32, and 21 days, respectively. Nine studies counted the time of viral shedding, which ranged from 1 to 32.6 days. Among them, there were 7 studies using integrated traditional Chinese and Western medicine regimens, and the time of viral shedding was 16, 1–6, 12.5, 23, 14.7, 4–21, and 7–23 days, respectively. There were 2 studies using TCM regimens alone, and the time of viral shedding was 15.2–32.6 and 19 days, respectively. One study counted the time to fever resolution, and the time was 1.76 days, and the treatment regimen was TCM treatment alone. One study counted the time of stool viral shedding, and the time was 15.5 days, and the treatment regimen was traditional Chinese and western medicine.

In the above studies, some of the time outcome indicators were calculated in the mean value, some in the median value, and the data cannot be combined and analyzed, so the results are presented in the form of tables. The details are presented in Table 1.

### Prospective Clinical Study

The 2 studies were published in 2021 and 2022, respectively. The detailed characteristics of the studies are presented in Table 2.

**TABLE 2:** Characteristics of 2 randomized control trials (RCTs) on children with COVID-19.

Study	Syndrome	Sex (M/F)	Age (Y)	Cases	Type (Mild/Moderate)	Treatment	Period (D)	Outcomes							
		T/C	T/C	T/C	T/C	T/C		Total effective rate (T/C)	Syndrome points	Fever disappearance rate	Cough disappearance rate	Immunoglobulin levels	Changes in the blood routine	Course (D)	ADRs
Li (43)	Cold-damp syndrome	17/13; 15/15	8.69 ± 2.37/ 9.23 ± 2.25	30/30	6/24; 9/21	Thunder-fire moxibustion plus Qingfei Paidu Decoction/Qingfei Paidu Decoction	5	83%/60%	The T was lower than C (*P* < 0.01)	No significant difference (*P* > 0.05)	The T was higher than C (*P* < 0.01)	The T was higher than C (P < 0.05 or *P* < 0.01)	No significant difference (*P* > 0.05)	−	No
Zhan et al. (42)	Damp-heat syndrome	31/19; 29/21	4.23 ± 3.15/ 5.33 ± 3.31	50/50	−	Yishen Jianpi massage combined with western medicine/western medicine	From admission to discharge	-	The T was lower than C (P < 0.05)	−	−	−	−	(3.76 ± 2.21)/(4.66 ± 2.18)	No

*“M,” male; “F,” female; “Y,” year; “D,” day; “T,” treatment group; “C,” control group; “ADRs,” adverse reactions; “−,” not mentioned in the original study.*

One study included 100 children with damp-heat syndrome of COVID-19. The treatment group used Yishen Jianpi massage combined with Western medicine, and the control group used Western medicine alone. The observation period was from admission to discharge. After treatment, symptom remission rate was higher in the treatment group than in the control group, and the mean disease duration of the two groups was 3.76 ± 2.21 and 4.66 ± 2.18 days, respectively. No adverse reactions occurred in either group. The study has shown that adding pediatric massage can shorten the course of the disease and relieve the symptoms (42).

One study included 60 children with cold-damp syndrome of COVID-19. The treatment group used thunder-fire moxibustion plus *Qingfei Paidu Decoction*, and the control group used *Qingfei Paidu Decoction* alone. After 5 days of treatment, cough disappearance rate and immunoglobulin levels were higher in the treatment group than in the control group, and there was no significant difference in fever disappearance rate. The study has shown that adding thunder-fire moxibustion can accelerate the improvement of part symptoms and improve body immunity (43).

**FIGURE 2:**
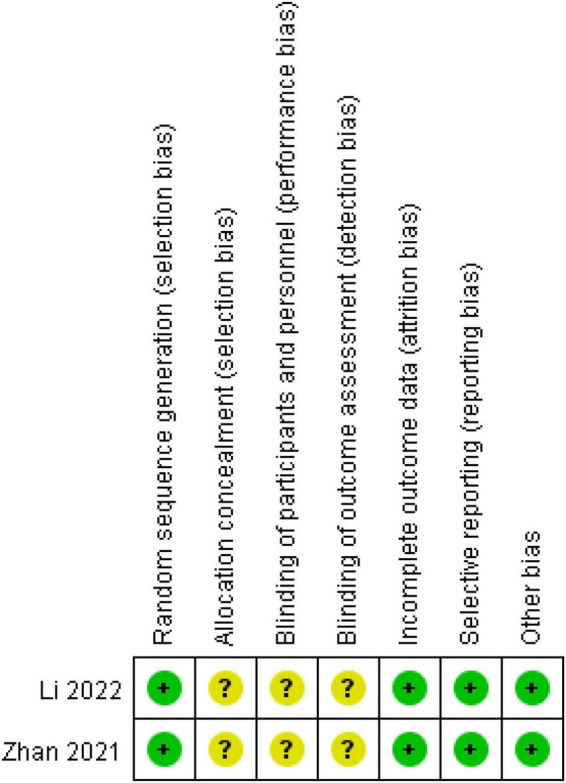
Assessment of risk of bias in 2 trials.

The assessment of risk of bias (Figure 2) showed that 2 studies implemented randomized grouping. There was no mention of allocation concealment and blinding. With regard to incomplete outcome data and selective reporting, the 2 studies reported the results according to preset outcome indicators and were rated as low risk. The 2 studies showed no other obvious biases and were rated as low risk.

The above results reflect the positive role of TCM external methods in the treatment of children with COVID-19. However, the above two studies only described the random number table method. No blinding methods and allocation concealment methods were mentioned. We contacted the corresponding author by telephone and email to seek a detailed study process without a response. Therefore, the results of the two randomized controlled studies have a lower evidence level.

## Discussion

According to the analysis of the above studies, TCM has a high participation rate in the treatment of COVID-19 in children. The external treatment methods also displayed their unique advantages. No obvious adverse effects appeared during the treatment process.

COVID-19 is a viral infectious disease. The survival of SARS-CoV-2 is related to environmental temperature and humidity (44–46). Different climates have different temperatures and humidity. Take the climate of southern and northern China as an example; the Qinling Mountains-Huaihe River is the dividing line between the north and the south of China, and between the warm temperate climate and the subtropical climate. Due to climatic differences, temperature and humidity are higher in the south than in the north. Therefore, it can lead to different disease characteristics. The most important feature of TCM is syndrome differentiation (47, 48). In the included studies above, damp-heat syndrome mostly appear in the south area (24, 38) and cold-damp syndrome mostly appear in the north area (25, 26). The main difference lies in the treatment of cold syndrome and heat syndrome. TCM theory has a treatment principle called “*Rezhe Hanzhi*, *Hanzhe Rezhi*” (cold prescriptions are used to treat the heat syndrome, and heat prescriptions are used to treat the cold syndrome). *Scutellaria baicalensis Georgi*, *Bupleurum Chinense (DC)*, and *gypsum* are the main representatives of heat-clearing medicine. Studies have shown that the antipyretic effect of *Scutellaria baicalensis Georgi* and *Bupleurum Chinense (DC)* is accomplished by regulating PGE2 and cAMP and inhibiting the synthesis or release of endogenous pyrogens TNF-α and β-EP (49, 50). The antipyretic effect of *gypsum* is mainly attributed to its trace elements, in which calcium, zinc, cadmium, cobalt, and lead directly participate in the antipyretic effect, and iron, copper and selenium play an indirect antipyretic role by regulating the immune system (51). *Ephedra sinica Stapf* and *Cinnamomi Ramulus Herb* are the main representatives of cold-dispelling medicine (52). *Luteolin*, the main active ingredient of *Ephedra sinica Stapf*, has strong anti-inflammatory, antiviral, and antibacterial effects, and it shows significant efficacy in treating severe acute respiratory syndrome (53). *Ephedra sinica Stapf* inhibited inflammatory response by reducing the amount of the airway inflammatory factors IL-3 and IL-4 in asthmatic rats (54). The antiviral effects of *Cinnamomi Ramulus volatile oil* and *Cinnamaldehyde* are reflected in reduction of proinflammatory cytokine release by regulating the proportion of T cell subsets, thus enhancing the immune defense system and alleviating lung tissue pathological damage (55). Cold and heat theory can be used as a bridge to understand the TCM theory.

Children of different age groups have different physiological and pathological characteristics. Children between 0 and 18 years old can be divided into baby period (birth to 1 year old), toddler period (1–3 years old), preschool period (3–7 years old), grade-schooler period (7–14 years old), and adolescent period (14–18 years old) (56–58). The studies above have shown that TCM can be used in the treatment of COVID-19 in children of all ages (23, 28, 33, 41), and that all children are cured and discharged from hospital, more or less, TCM treatment regimens have been added. The curative effect of TCM is certain, and no obvious adverse reactions occurred. The treatment of children with COVID-19 is still inexperienced. In the future, child cases should be collected in time, and treatment regimens should be continuously summarized and improved (59–61). TCM participates in the treatment of COVID-19 in children of all ages, which is a positive option in clinical practice.

Moreover, external treatment methods provide more options for the treatment of COVID-19 in children, and the advantages of external treatment methods have been gradually shown (62). External methods for children mainly include massage (63), acupuncture (64), herbal plaster (65), and so on. External treatment is mostly operated in the body surface, which can observe the patient’s tolerance situation at any time, thus deciding whether to continue the treatment. Although the two studies included had lower levels of evidence, they provide a reference for external treatment of children with COVID-19. More high-quality clinical and experimental research should be carried out in the future.

At present, China is actively introducing to the world a continuous improvement practice experience in COVID-19 TCM diagnosis and treatment. The main measures include remote video exchange of treatment experience, donation of TCM materials, and support of TCM doctors to foreign countries. However, because of differences in national policies, systems, and cultures, the spread of TCM is limited to some extent (65). The number of English media reports on TCM has increased, and the global English audience has become more familiar with TCM, which has built a positive image of TCM (66). At present, the evidence of efficacy and safety of TCM treatment of COVID-19 is increasing. Therefore, the key point for TCM to be recognized is efficacy. Our study further adds evidence to the treatment of COVID-19 in children using TCM.

There are also some limitations to our study. Most of the included literature is retrospective studies, most of the studies have fewer cases, and the outcome indicators are calculated with different methods, which cannot objectively compare the advantages of TCM in treating COVID-19 in children. The included RCT studies were partially methodologically lacking, and there are no RCT studies treatment with oral TCM drugs. Therefore, more high-quality, well-designed, multicenter RCT studies are still needed to provide evidence for the efficacy and safety of TCM in treating children with COVID-19 in the future.

## Conclusion

At present, TCM is widely used in treatment of children with COVID-19, and no obvious adverse reactions have occurred. Application of TCM is a positive option in clinical practice. However, most of the current studies are retrospective clinical observational studies, and the randomized controlled studies have lower levels of evidence. More high-level evidence is still needed to verify the effectiveness and safety of TCM treatment for COVID-19 in children.

## Author Contributions

CL, ND, and BL conceived and designed the review. ND, XL, and BL wrote the initial draft. YX, YM, and LL were responsible for literature checking and result discussion. All authors contributed to the final version of the manuscript.

## Conflict of Interest

The authors declare that the research was conducted in the absence of any commercial or financial relationships that could be construed as a potential conflict of interest. The reviewer HT declared a shared affiliation with the authors at the time of review.

## Publisher’s Note

All claims expressed in this article are solely those of the authors and do not necessarily represent those of their affiliated organizations, or those of the publisher, the editors and the reviewers. Any product that may be evaluated in this article, or claim that may be made by its manufacturer, is not guaranteed or endorsed by the publisher.

## Abbreviations

COVID-19: Coronavirus disease 2019
SARS-CoV-2: severe acute respiratory syndrome coronavirus 2
TCM: Traditional Chinese Medicine
WHO: World Health Organization
RCT: randomized controlled trial.
